# Expression of the NUP153 and YWHAB genes from their canonical promoters and alternative promoters of the LINE-1 retrotransposon in the placenta of the first trimester of pregnancy

**DOI:** 10.18699/VJGB-23-09

**Published:** 2023-03

**Authors:** V.V. Demeneva, E.N. Tolmacheva, T.V. Nikitina, E.A. Sazhenova, S.Yu. Yuriev, A.Sh. Makhmutkhodzhaev, A.S. Zuev, S.A. Filatova, A.E. Dmitriev, Ya.A. Darkova, L.P. Nazarenko, I.N. Lebedev, S.A. Vasilyev

**Affiliations:** Research Institute of Medical Genetics, Tomsk National Research Medical Center of the Russian Academy of Sciences, Tomsk, Russia; Research Institute of Medical Genetics, Tomsk National Research Medical Center of the Russian Academy of Sciences, Tomsk, Russia; Research Institute of Medical Genetics, Tomsk National Research Medical Center of the Russian Academy of Sciences, Tomsk, Russia; Research Institute of Medical Genetics, Tomsk National Research Medical Center of the Russian Academy of Sciences, Tomsk, Russia; Siberian State Medical University, Tomsk, Russia; Siberian State Medical University, Tomsk, Russia; Research Institute of Medical Genetics, Tomsk National Research Medical Center of the Russian Academy of Sciences, Tomsk, Russia; National Research Tomsk State University, Tomsk, Russia; National Research Tomsk State University, Tomsk, Russia; National Research Tomsk State University, Tomsk, Russia; Research Institute of Medical Genetics, Tomsk National Research Medical Center of the Russian Academy of Sciences, Tomsk, Russia; Research Institute of Medical Genetics, Tomsk National Research Medical Center of the Russian Academy of Sciences, Tomsk, Russia Siberian State Medical University, Tomsk, Russia; Research Institute of Medical Genetics, Tomsk National Research Medical Center of the Russian Academy of Sciences, Tomsk, Russia National Research Tomsk State University, Tomsk, Russia

**Keywords:** miscarriage, placenta, retrotransposon LINE-1, DNA methylation, NUP153, YWHAB, невынашивание беременности, плацента, ретротранспозон LINE-1, метилирование ДНК, NUP153, YWHAB

## Abstract

The placenta has a unique hypomethylated genome. Due to this feature of the placenta, there is a potential possibility of using regulatory elements derived from retroviruses and retrotransposons, which are suppressed by DNA methylation in the adult body. In addition, there is an abnormal increase in the level of methylation of the LINE-1 retrotransposon in the chorionic trophoblast in spontaneous abortions with both normal karyotype and aneuploidy on different chromosomes, which may be associated with impaired gene transcription using LINE-1 regulatory elements. To date, 988 genes that can be expressed from alternative LINE-1 promoters have been identified. Using the STRING tool, genes (NUP153 and YWHAB) were selected, the products of which have significant functional relationships with proteins highly expressed in the placenta and involved in trophoblast differentiation. This study aimed to analyze the expression of the NUP153 and YWHAB genes, highly active in the placenta, from canonical and alternative LINE-1 promoters in the germinal part of the placenta of spontaneous and induced abortions. Gene expression analysis was performed using real-time PCR in chorionic villi and extraembryonic mesoderm of induced abortions (n = 10), adult lymphocytes (n = 10), spontaneous abortions with normal karyotype (n = 10), and with the most frequent aneuploidies in the first trimester of pregnancy (trisomy 16 (n = 8) and monosomy X (n = 6)). The LINE-1 methylation index was assessed in the chorionic villi of spontaneous abortions using targeted bisulfite massive parallel sequencing. The level of expression of both genes from canonical promoters was higher in blood lymphocytes than in placental tissues (p < 0.05). However, the expression level of the NUP153 gene from the alternative LINE-1 promoter was 17 times higher in chorionic villi and 23 times higher in extraembryonic mesoderm than in lymphocytes (p < 0.05). The expression level of NUP153 and YWHAB from canonical promoters was higher in the group of spontaneous abortions with monosomy X compared to all other groups (p <0.05). The LINE-1 methylation index negatively correlated with the level of gene expression from both canonical (NUP153 – R = –0.59, YWHAB – R = –0.52, p < 0.05) and alternative LINE-1 promoters (NUP153 – R = –0.46, YWHAB – R = –0.66, p < 0.05). Thus, the observed increase in the LINE-1 methylation index in the placenta of spontaneous abortions is associated with the level of expression of the NUP153 and YWHAB genes not only from alternative but also from canonical promoters, which can subsequently lead to negative consequences for normal embryogenesis.

## Abstract (ru)

Для плаценты характерен уникальный гипометилированный геном. Благодаря этой особенности плаценты в первом триместре беременности наблюдается потенциальная возможность использования регуляторных элементов, полученных от ретровирусов и ретротранспозонов, которые во взрослом организме подавляются метилированием ДНК. Кроме того, у спонтанных абортусов и с нормальным кариотипом, и с анеуплоидиями отмечается аномальное повышение уровня метилирования ретротранспозона LINE-1 в трофобласте хориона, что может быть связано с нарушением транскрипции генов с использованием регуляторных элементов LINE-1. На сегодняшний день идентифицировано 988 генов, способных экспрессироваться с альтернативных промоторов LINE-1. Из них с помощью инструмента STRING были отобраны гены, продукты которых взаимодействуют с белками, экспрессирующимися на высоком уровне в плаценте и участвующими в дифференцировке трофобласта, NUP153 и YWHAB. Целью настоящего исследования являлся анализ экспрессии генов NUP153 и YWHAB с канонических и альтернативных промоторов ретротранспозона LINE-1 в зародышевой части плаценты спонтанных и медицинских абортусов первого триместра беременности. Определение уровня экспрессии генов проводили с помощью ПЦР в режиме реального времени в лимфоцитах взрослых индивидов (n = 10), в ворсинах хориона и экстраэмбриональной мезодерме медицинских абортусов (n = 10) и спонтанных абортусов с нормальным кариотипом (n = 10) и с наиболее частыми анеуплоидиями в I триместре беременности (трисомия 16 (n = 8) и моносомия X (n = 6)). Индекс метилирования LINE-1 оценивали в ворсинах хориона спонтанных абортусов с помощью таргетного бисульфитного массового параллельного секвенирования. Уровень экспрессии обоих генов с канонических промоторов был выше в лимфоцитах крови, чем в тканях плаценты (p < 0.05). Однако уровень экспрессии гена NUP153 с альтернативного промотора LINE-1 был выше в 17 раз в ворсинах хориона и в 23 раза – в экстраэмбриональной мезодерме по сравнению с лимфоцитами (p < 0.05). Между группами спонтанных абортусов с моносомией X и остальными группами были выявлены статистически значимые различия. Уровень экспрессии NUP153 и YWHAB с канонических промоторов был выше в группе спонтанных абортусов с моносомией X по сравнению со всеми другими группами (p < 0.05). Индекс метилирования LINE-1 отрицательно коррелировал с уровнем экспрессии генов как c канонических (NUP153 – R = –0.59, YWHAB – R = –0.52, p < 0.05), так и c альтернативных промоторов LINE-1 (NUP153 – R = –0.46, YWHAB – R = –0.66, p < 0.05). Таким образом, наблюдаемое нами повышение индекса метилирования LINE-1 в плаценте спонтанных абортусов связано с уровнем экспрессии генов NUP153 и YWHAB не только с альтернативных, но и с канонических промоторов, что в дальнейшем может приводить к негативным последствиям для нормального эмбриогенеза.

## Introduction

In humans, reproductive losses are more common in the first trimester of pregnancy than in other periods of embryogenesis. One of the most common causes of early embryonic death is an abnormal number of chromosomes (aneuploidy), which leads to severe developmental anomalies. The formation of aneuploidy with meiotic and mitotic origin corresponds to the waves of epigenetic reprogramming, in particular, genome demethylation in the zygote and at the cleavage stage. Early blastocyst demonstrates less DNA methylation at the latter stage than cells at any other moment of ontogeny (Smith et al., 2012). A rapid wave of de novo DNA methylation for the inner cell mass then follows while the trophectoderm remains hypomethylated (Santos et al., 2010).

Throughout pregnancy, the placenta has a unique hypomethylated epigenetic landscape compared to other extraembryonic and embryonic tissues, which may indicate its special functions (Robinson, Price, 2015). Hypomethylation in placental DNA occurs mainly in “partially methylated domains” and is unevenly distributed throughout the genome. “Partially methylated domains” refers to large (> 100 kb) regions of low DNA methylation alternating with regions of higher DNA methylation (Schroeder et al., 2013).

The placenta exhibits reduced DNA methylation of some types of repetitive genome elements (Price et al., 2012). One of them, the LINE-1 retrotransposon (long interspersed nuclear element-1), is the largest, occupying approximately 20 % of the genome, and the most evolutionarily young class of retrotransposons in humans, retaining the ability to transpose (Ostertag et al., 2001). The transcriptional activity of LINE-1 is suppressed by DNA methylation during most periods of ontogeny.

An important feature of LINE-1 that requires attention is its high level of methylation in blood leukocytes, regardless of age and gender, while the level of LINE-1 methylation in other tissues has its tissue-specific differences (Chalitchagorn et al., 2004). It was shown that for the placenta as an independent organ, the level of methylation of retrotransposons doesn’t always coincide with the global level of methylation of the entire genome. The level of LINE-1 methylation in the tissues of the placenta of the third trimester of pregnancy significantly decreases compared to the first trimester of pregnancy. At the same time, changes in the DNA methylation level of the entire genome are not found between the first and third trimester placentas (He et al., 2014).

It can be assumed that LINE-1 methylation and activation are transiently regulated during normal placental development. This raises the question of a possible functional role for LINE-1 retrotransposon sequences in placental development. Previously, we found that some spontaneous abortions with normal karyotypes were characterized by epigenetic disorders similar to spontaneous abortions with aneuploidy. In particular, some spontaneous abortions with a normal karyotype had an increased methylation index in the LINE-1 retrotransposon promoter, which was characteristic of groups of spontaneous abortions with trisomy 16 and monosomy X (Vasilyev et al., 2021b).

One of the possible roles of LINE-1 may be the usage of its regulatory sequences to influence the transcription of adjacent genes. This effect becomes feasible because LINE-1 includes a sense promoter that controls the transcription of the ORF1 and ORF2p proteins required for retrotransposition, and an antisense promoter that controls the transcription of chimeric transcripts, LINE-1 5′-antisense sequences spliced with exons of neighboring genes (Denli et al., 2015). LINE-1 antisense transcripts affect up to 4 % of all human genes, and LINE-1 antisense promoters are actively transcribed in various types of human cells, including embryonic tissues. A total of 988 genes that can be expressed from alternative LINE-1 promoters have been identified so far (Criscione et al., 2016b). It is possible that the expression of multiple genes in extraembryonic tissues may occur predominantly from alternative LINE-1 promoters because LINE-1 promoters are hypomethylated in the placenta.

**Fig. 1.**
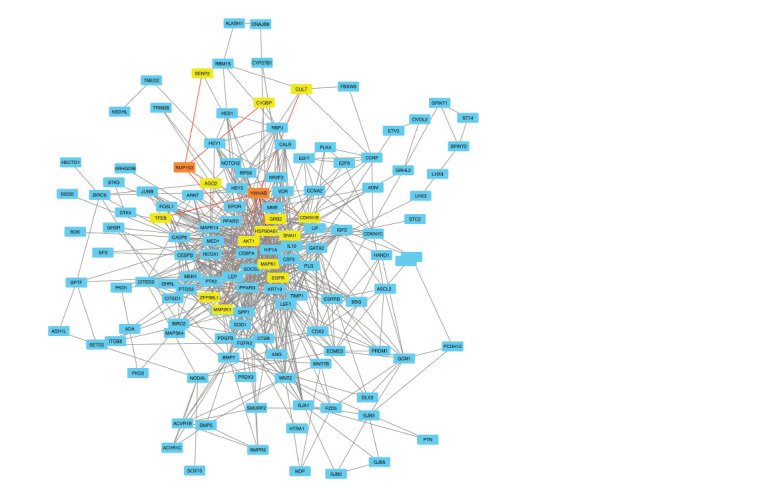
Functionally significant connections of the proteins involved in the development of the placenta (GO:0061450, GO:0097360, GO:0001890) with the NUP153 and YWHAB genes according to STRING Yellow shows proteins that have functional bonds (highlighted in red) with the NUP153 and YWHAB genes (marked in orange) (STRING score > 0.4).

Using the STRING tool, two genes, NUP153 and YWHAB, were selected among the genes capable of expression from alternative LINE-1 promoters. Their products showed a high level of expression in the placenta and are functionally associated with proteins involved in trophoblast differentiation (according to Gene Ontology: GO:0061450, trophoblast cell migration; GO:0097360, chorionic trophoblast cell proliferation; GO:0001890, placenta development) (Fig. 1). The NUP153 gene functions as a scaffolding element in the nuclear phase of the nuclear pore complex. It is required for normal nuclearcytoplasmic transport of proteins and mRNA during somatic cell division (Bilir et al., 2019) and in mouse embryonic stem cells (Souquet et al., 2018). The YWHAB gene belongs to the group of genes responsible for signal transduction by binding to phosphoserine-containing proteins. The protein encoded by the gene interacts with RAF1 and CDC25 phosphatases and may play a role in mitogenic signaling and cell cycle regulatory mechanisms. It was shown that YWHAB overexpression stimulates and maintains attachment-independent cell growth in a fibroblast cell line isolated from mouse embryos (Sasaki et al., 2014).

The aim of this study was to analyze the expression of the NUP153 and YWHAB genes from canonical and alternative LINE-1 promoters in the germinal part of the placenta of spontaneous and induced abortions.

## Materials and methods

The samples were from chorionic villi and extraembryonic mesoderm of induced abortions (IA) (n = 10, gestational age 8.2 ± 2.3 weeks), spontaneous abortions (SA) with normal karyotype (n = 10, gestational age 7.2 ± 1.4 weeks), trisomy 16 (n = 8, gestational age 6.5 ± 0.8 weeks), and monosomy X (n = 6, gestational age 8.6 ± 0.7 weeks). Samples were taken from the Biobank of Northern Eurasia of the Research Institute of Medical Genetics of the Tomsk National Research Medical Center. The samples were obtained from 2004 to 2021 and stored in liquid nitrogen, before their use for analysis. The study was conducted in compliance with ethical standards by the Helsinki Declaration of the World Medical Association. The study was approved by the Biomedical Ethics Committee of the Research Institute of Medical Genetics of the Tomsk National Research Medical Center (November 9, 2020/No. 7).

A standard cytogenetic analysis was performed on direct preparations of chorionic villi and fibroblast cultures of the extraembryonic mesoderm to determine the karyotype (Lebedev et al., 2004). Karyotyping results for 14 trisomic and monosomic SA samples were confirmed by fluorescence in situ hybridization (FISH). Aneuploidy mosaicism was assessed with a lower cutoff of 10 % and an upper cutoff of 90 %.

Centromere-specific DNA probes for X chromosomes were used for the analysis of monosomy X and subtelomeric DNA probes (16q and 16p) were used for the analysis of trisomy 16. The analysis was carried out according to the protocol described elsewhere (Vasilyev et al., 2010). Four samples had a mosaic karyotype with a trisomy level from 10 to 90 %. The remaining 10 spontaneous abortions with a higher proportion of trisomy or monosomy were classified as having pure aneuploidy. The blood lymphocytes of IA parents (5 couples, age 30.8 ± 2.7) were used as a comparison group that were contained in the Lyra reagent (Biolabmix, Russia) before the start of the experiment.

RNA was isolated from chorionic villi and extraembryonic mesoderm by the phenol-chloroform method. All tissues were stored in liquid nitrogen from the moment of obtaining the material of the studied samples to the beginning of RNA isolation. Tissue separation was preliminarily carried out in RNAlater (Invitrogen, USA) to stabilize the RNA in the samples. Each sample was homogenized in a mortar with liquid nitrogen, adding 500 μl of Lyra reagent (Biolabmix, Russia). The lysate was incubated first for 5 min at 55 °C, then for 5 min at room temperature. The lysate was then centrifuged at 12,000 rpm for 10 min to remove undissolved fragments, and the supernatant was transferred into a new tube. A volume of 0.2 ml of chloroform was added per each 1 ml of Lyra reagent, followed by shaking (by hand) for 15 s, followed by incubation of the mixture for 10 min at room temperature, and centrifugation at 10,000 g for 10 min at 4 °C. Next, 0.5 ml of 100 % cold isopropanol was added to the aqueous phase containing RNA per each 1 ml of Lyra reagent, and the mixture was incubated at –20 °C for 10 min, after which the sample was centrifuged at 12,000 g for 10 min at 4 °C.

The precipitate was washed twice with 80 % cold ethanol at 10,000 g for 5 min at 4 °C. The precipitate was then dried for 2 min in a concentrator (Eppendorf, USA) (parameters: 45 °C, V-AL). After this, 40 μl of DEPC water and 1 μl of RiboLock (Thermo, USA) were added to dissolve the precipitate and left for 10 min at room temperature until complete dissolution. All samples were kept on ice to avoid RNA degradation during isolation whereas at the incubation stage all steps were performed at room temperature. All samples were stored at –80 °C after isolation

The RNA was treated with DNase (Biolabmix) to obtain pure RNA. Further, the OT-M-MuLV-RH kit (Biolabmix) with a random hexaprimer was used for reverse transcription. The reverse transcription reaction mixture included 1.5 μg RNA, 3 μl hexaprimer, 4 μl KCl reaction buffer, 2 μl 0.1 M DDT, 1 μl 10 mM dNTP mix, and 1 μl revertase. Two types of primers were designed for the NUP153 and YWHAB genes: the first for long products that are expressed only from canonical promoters, and the second for short products that are expressed from alternative LINE-1 antisense promoters (see the Table).

**Table 1.**
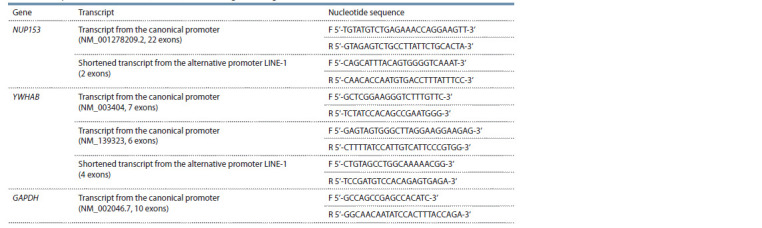
Sequences of oligonucleotide primers for assessing the level of expression of the NUP153, YWHAB, and GAPDH genes using real-time PCR Notе. F– forward primer; R – reverse primer.

**Fig. 2.**
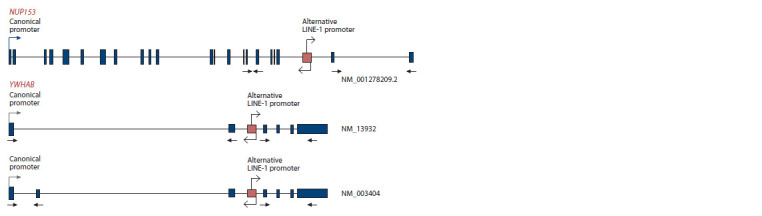
Scheme of the location of alternative LINE-1 promoters for the NUP153 and YWHAB genes The arrows schematically mark the hybridization sites of oligonucleotide primers. The arrow starting at the beginning of the LINE-1 element indicates the direction of transcription from the direct LINE-1 promoter, which is canonical. The second arrow pointing in the opposite direction marks the direction of expression from the alternative antisense LINE-1 promoter, which is also alternative for the studied genes.

The NUP153 gene includes 22 exons, while the short transcript from the alternative LINE-1 promoter contains only exons 21–22. Primers were designed in exons 16–17 for detecting NUP153 gene transcripts from the canonical promoter and in exons 21–22 for detecting transcripts from the alternative promoter. Two normal long transcripts with exons 7 or 6 are transcribed from the normal promoter of the YWHAB gene. In this regard, primers were designed for each product. For the first transcript with seven exons, primers were designed in exons 1–2. For the second transcript with 6 exons, primers were designed in exons 1–3. Primers of the short product from the alternative LINE-1 promoter of the YWHAB gene were designed in exons 4–7 (Fig. 2). The expression from alternative gene promoters was taken to be the difference between the level of gene expression estimated using primers specific to the region downstream the alternative promoter and the level of gene expression estimated using primers annealing upstream in the first exons. This value was used for data analysis and is displayed on the charts. For the YWHAB gene, the sum of expression levels of both long transcripts was subtracted from the expression level of the canonical promoter.

The methylation index was assessed in 19 CpG sites of the LINE-1 promoter in chorionic villi of spontaneous abortions using targeted bisulfite massive parallel sequencing. Library preparation and evaluation were carried out according to a previously published protocol (Vasilyev et al., 2021a). Statistical analysis of data was performed using Statistica 10.0 software.

## Results

**Fig. 3.**
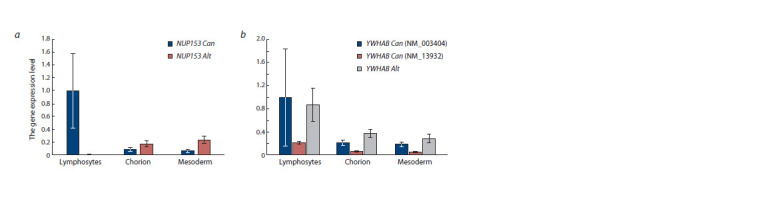
Comparison of the NUP153 (a) and YWHAB (b) gene expression levels from canonical promoters and alternative LINE-1 promoters in blood lymphocytes, chorion, and placental mesoderm. Values are given as fold differences relative to the level of gene expression from the canonical promoter in adult lymphocytes. Expression levels of two different transcripts from the canonical promoter (NM_003404, NM_139323) are shown for the YWHAB gene. The reference gene is GAPDH. Can is the canonical promoter, and Alt is the alternative LINE-1 promoter.

**Fig. 4.**
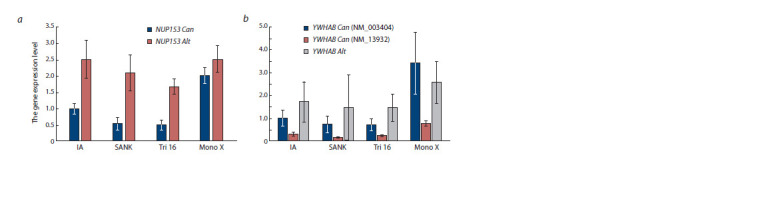
Comparison of the expression level of the NUP153 (a) and YWHAB (b) genes from canonical promoters and alternative LINE- 1 promoters between groups of spontaneous abortions and induced abortions. Values are given as fold differences relative to the level of gene expression of the canonical promoter in the group of induced abortions. SANK – spontaneous abortions with normal karyotype; Tri 16 – spontaneous abortions with trisomy 16; Mono X – spontaneous abortions with monosomy X.

The expression level of the NUP153 gene from the canonical promoter was 12.5 times higher in lymphocytes than in placental tissues ( p = 0.000001). The expression level of the YWHAB gene from the canonical promoter was also on average higher in blood lymphocytes than in placental tissues (by 4.6 times) (transcript NM_13932 ( p = 0.00003)). The expression level of the NM_003404 transcript of the YWHAB gene was highly variable in lymphocytes. However, the expression level of the NUP153 gene from alternative LINE-1 promoters was statistically significantly higher in extraembryonic tissues compared to lymphocytes of adults (17 times in chorionic villi and 23 times in extraembryonic mesoderm, p < 0.05) (Fig. 3). The levels of expression of both genes from canonical promoters were higher in the SA group with monosomy X than in the groups of SA with normal karyotype (Fig. 4).

**Fig. 5.**
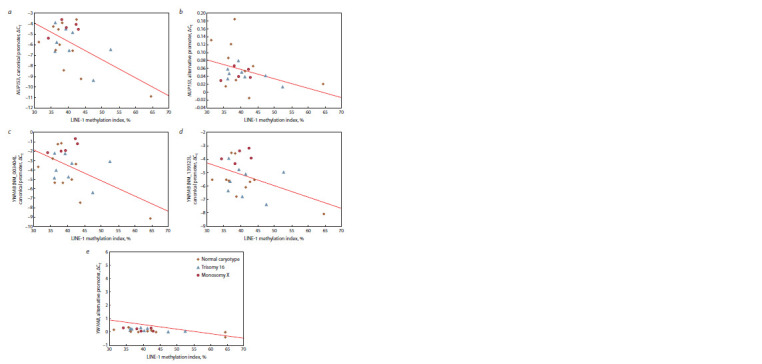
Correlation of the NUP153 and YWHAB gene expression with the LINE-1 methylation index in the chorionic trophoblast of spontaneous abortions with normal karyotype, trisomy 16, and monosomy X. Correlations of the LINE-1 methylation index with various transcripts of the NUP153 and YWHAB genes: a – NUP153 gene expression from the canonical promoter; b – NUP153 gene expression from the alternative promoter; c – YWHAB (NM_003404) gene expression from the canonical promoter; d – YWHAB (NM_139323) gene expression from the canonical promoter; e – YWHAB gene expression from the alternative promoter. SANK – spontaneous abortions with normal karyotype; Tri 16 – spontaneous abortions with trisomy 16; Mono X – spontaneous abortions with monosomy X.

The level of methylation of the LINE-1 retrotransposon promoter was assessed in the chorionic villi of spontaneous abortions with different karyotypes. The average level of LINE-1 methylation in chorionic villi of SA was 41.9 ± ± 5.8 % with trisomy 16, 39.7 ± 3.6 % with monosomy X, and 38.4 ± 3.9 % with normal karyotype. The LINE-1 methylation index negatively correlated with the level of gene expression from both canonical (NUP153 – R = –0.59, p < 0.003; YWHAB – R = –0.52, p < 0.01) and alternative LINE-1 promoters (NUP153 – R = –0.46, p = 0.03; YWHAB – R = –0.66, p = 0.001) (Fig. 5).

## Discussion

In the present work, it was found that the level of expression of the NUP153 and YWHAB genes in the placenta from canonical promoters was lower compared to the adult blood lymphocytes, but the expression of the NUP153 gene from the alternative LINE-1 promoter was higher in the placenta. This result has supported the hypothesis that in the placenta, the expression of genes from alternative promoters derived from retroviruses and retrotransposons can be activated due to the hypomethylated epigenetic landscape. This assumption is also supported by the enrichment of genes that are tissue-specifically expressed in the placenta among all genes which can be transcribed from alternative LINE-1 promoters (Criscione et al., 2016a).

We have not found significant differences in the level of expression of the YWHAB and NUP153 genes from alternative promoters between groups of spontaneous abortions with different karyotypes and the control group of induced abortions. At the same time, the levels of expression of both genes from canonical promoters were higher in the group of spontaneous abortions with monosomy X. However, it has been found that the level of expression of the studied genes changes in individual spontaneous abortions depending on changes in the level of LINE-1 methylation. The obtained data clearly demonstrated that the expression level of the NUP153 and YWHAB genes from the canonical and alternative LINE-1 promoters correlates with the LINE-1 methylation level: the higher the LINE-1 methylation level, the lower the expression

There can be several reasons for the relationship between the level of LINE-1 methylation and the expression of the studied genes from both promoters. First, a short transcript from an alternative promoter may be associated with the activation of gene transcription from the canonical promoter. However, this option seems unlikely, because the expression level of the studied genes from canonical promoters against the background of genome hypomethylation in the placenta was lower than in lymphocytes, which are characterized by a LINE-1 methylation level of more than 70 % (Rosser, An, 2012). This should be the opposite if this hypothesis is correct. Second, the level of methylation of the LINE-1 retrotransposon may reflect the global level of genome methylation and the level of methylation in the canonical promoter of the studied genes. This variant seems to be more likely, but also doesn’t remove the issue of reduced expression of the studied genes in the placenta against the background of a hypomethylated epigenetic landscape compared to adult lymphocytes. The expression of the studied genes is regulated not only by methylation but also by tissue-specific transcription factors.

It remains unclear whether the NUP153 and YWHAB gene expression both from the canonical and alternative promoter plays a functional role in the placenta or whether these transcripts are by-products of the genome hypomethylation. Potentially, the impaired NUP153 expression can have a negative impact on the nuclear-cytoplasmic transport of proteins and mRNA, and the abnormal YWHAB gene expression can affect the transmission of cell signals.

NUP153 and YWHAB gene products have significant functional connections with proteins involved in the differentiation of the trophoblast (see Fig. 1). NUP153 interacts with the AGO2, SENP2, C1QBP, and PPARD genes. A list of significant connections is wider for the YWHAB gene – it interacts with the TFEB, CUL7, ZFP36L, MAP2K1, AKT1, CDKN1B, SNAI1, MAPK1, and EGFR genes

The impaired function of each of these genes has a negative effect on the normal course of embryogenesis. For example, the normal expression of MAPK1 is necessary for the development of non-embryonic ectoderm during placentogenesis. Its absence can lead to embryo death due to abnormal development and hypovascularization of the placenta (Bissonauth et al., 2006). The CUL7 gene is actively expressed in the cell lines of the trophoblast. Protein deficiency of the CUL7 gene is associated with a delay in intrauterine development due to abnormal development of the placenta, which leads to intrauterine hypoxia (Fahlbusch et al., 2012). The deficit can lead to the occurrence of cutaneous or hypodermal hemorrhages, as well as the development of trophoblast with abnormal vascular structure at later stages of gestation (Arai et al., 2003). CUL7 mutations in the embryo line are associated with the 3-M syndrome, which is characterized by pre- and postnatal growth retardation (Maksimova et al., 2007; Fu et al., 2010).

The SENP2 gene belongs to the family of ubiquitin-like proteins and is localized in the cell in the nuclear pores and cytoplasm (Talamillo et al., 2020). SENP2 mutations impair cell cycle progression during trophoblast development in mice: deletion of SENP2 impairs the p53/Mdm2 pathway, affecting trophoblast progenitor cells and their maturation (Chiu et al., 2008). SENP2 influences the normal development of cardiomyocytes during further differentiation. Overexpression causes abnormal proliferation of cardiomyocytes with dysregulation of cyclin and cyclin-dependent kinase inhibitors, leading to congenital heart anomalies (Kim et al., 2012). On the other hand, deletions also cause defects in myocardial development due to reduced proliferation (Kang et al., 2010).

It is logical to assume that the existing functional relationships of the NUP153 and YWHAB genes with genes involved in trophoblast differentiation can go both in a negative direction and in a protective one. Pathological changes in the expression of the NUP153 and YWHAB genes can potentially lead to impaired function of other genes, the formation of a pathological embryo phenotype, or even embryonic death.

## Conclusion

We have revealed that the NUP153 and YWHAB genes in the placenta tissues are predominantly expressed from alternative LINE-1 promoters located in the intrones. Even though the expression from alternative promoters of LINE-1 was higher than with canonical gene promoters for all groups (spontaneous and induced abortions), and there were no significant differences in the level of expression of the YWHAB and NUP153 genes from alternative promotors between groups, we have seen a trend towards the general decrease in expression in spontaneous abortions compared to induced abortions. However, it has been found that the level of expression of the studied genes changes in individual spontaneous abortions, depending on changes in the level of genome methylation. The obtained data demonstrate the relationship between the levels of the NUP153 and YWHAB gene expression from canonical and alternative LINE-1 promoters with LINE-1 methylation levels in extraembryonic tissues of spontaneous abortions.

Thus, an increase in the LINE-1 methylation index in the placenta of spontaneous abortions may be associated with a decrease in gene expression not only from alternative but also from canonical promoters. The revealed features of the relationship between the LINE-1 methylation level with the NUP153 and YWHAB gene expression levels indicate an existing mechanism for self-regulation of normal embryogenesis, disturbance of which can lead to embryo death.

## Conflict of interest

The authors declare no conflict of interest.
